# Study of Heat Treatment Effect on Mechanical Properties of Epoxy Resin Reinforced with Fiber Glass

**DOI:** 10.3390/polym15122734

**Published:** 2023-06-19

**Authors:** Zhenbo Lan, Jiangang Deng, Zhuolin Xu, Zhu Ye, Yu Nie

**Affiliations:** 1 Wuhan Nari Limited Liability Company of State Grid Electric Power Research Institute, Wuhan 430000, China; lan15926378682@163.com (Z.L.); dengkelvin@163.com (J.D.); 15872380396@139.com (Z.Y.); whnr18327057305@163.com (Y.N.); 2 State Grid Electric Power Research Institute, Nanjing 210000, China

**Keywords:** epoxy resin, glass fiber, thermal aging, mechanical properties, failure mechanism

## Abstract

In this paper, mechanical properties of the diglycidyl ether of bisphenol A epoxy resin (EP) reinforced with a 20% fiber glass (GF) with layered structure after high temperature aging are studied. Tensile and flexural stress–strain curves of the GF/EP composite after aging tests in the temperature range of 85–145 °C in air were measured. Tensile and flexural strength demonstrate gradual decrease with the increase in the aging temperature. The failure mechanism at the micro scale is studied by the scanning electron microscopy. A separation of the GFs and EP matrix and evident pullout of the GFs are observed. Degradation of the mechanical properties is explained by a cross-linking and chain scission of the initial molecular structure of the composite and decrease in the interfacial adhesion force between GFs and EP matrix caused by oxidation of the EP matrix and difference of the GF and EP coefficients of thermal expansion.

## 1. Introduction

High-molecular-weight epoxy resin (EP) has a few epoxy groups which react with a curing agent forming a 3D thermosetting network [1]. During the aging processes, EP mechanical properties and appearance (yellowing and cracking) are deteriorated, shortening its service life [2,3]. Aging factors which influence the service life can be sorted into two groups of external and internal effects. The parameters of the material itself influencing the service life are the internal factors, including structure and composition of the material; impurities in the material can also play a considerable role [4,5]. The parameters of the external conditions are temperature, humidity, atmosphere composition, electric field, electromagnetic and high-energy particle irradiation. These parameters have strong effect influencing the internal characteristics of the material and result in its aging [6,7,8]. During aging, internal and external factors result in change in the molecular chain structure (branching, breaking) of the EP, which leads to deterioration of mechanical and other properties.

If the EP is in a high-temperature oxygen atmosphere, its molecular chains can absorb the external oxygen and form hydroperoxide [7]. Due to the fact that peroxides are unstable, restructuring of the polymer occurs, including cross-linking and breaking of the chains. Such restructuring leads to deterioration of the EP properties due to thermal oxygen aging. During use, storage, and processing, the EP is often subjected to high-temperature stresses. When the temperature increases, at a certain value, thermal oxygen aging starts [9]. Thermal oxidation features of EP at different temperatures were studied by Lin X. et al. [10]. At the beginning of aging treatment, the shear strength of the EP is increased. This effect can be explained by increasing curing degree of the resin due to post-curing process. Further, after the curing is completed, the shear strength starts to decrease due to oxidative aging of the EP. Tang et al. used the Kissinger method to study the thermal decomposition characteristics of the EP cured with different curing systems at different heating rates [11]. The results showed that under nitrogen atmosphere, the EP cured with diethylenetriamine and m-phenyldimethylamine as curing agents was subjected to a two-stage pyrolysis process. The cured EP using low-molecular-weight polyamide (PA10) as the curing agent is least affected by the heating rate, but as the heating rate increases, the two-stage pyrolysis gradually transforms to the tertiary pyrolysis. In a humid environment, the EP is subjected to the damp heat aging process [12], which also deteriorates the performance of the EP. The damp heat aging results in microcraking, spallation at the interface in the EP composites, and post-heating curing of the EP [8,13,14]. Webster et al. synthesized a novel vinyl ether monomer from eugenol, a 2-eugenoloxyvinyl ether, which was copolymerized with cyclohexyl vinyl ether (CHVE). The epoxidized co-polymers showed an increase in thermal stability from 213 to 241 °C depending on content of the CHVE [15].

Therefore, a long-term treatment at high temperature inevitably results in EP aging, which is manifested as degradation of mechanical properties and loss of adhesion. One of the ways to solve the problem of high-temperature degradation is adding fiber fillers during the EP curing. Widely used synthetic fiber fillers are carbon fibers, glass wool, aramid, mineral wool, and glass fibers. After reinforcement with a relatively low fraction of fiber fillers, polymers with crosslinked structures demonstrate improvement in their mechanical (tensile stress and elastic modulus), high-temperature, and corrosive performance [16,17]. The EPs reinforced with fiber fillers are often used in many industrial fields such as electronic and aerospace equipment, coatings and adhesives [18,19,20,21].

High-temperature aging in oxygen environment of the fiber-reinforced bisphenol EP cured by anhydride was studied by Yang et al. [22]. During thermal aging in an oxygen environment, infra-red (IR) spectrometry revealed molecular structure reconstruction and oxidation in the EP surface layer. It was shown by dynamic thermal analysis that the structure reconstruction during oxidation takes place in the thin (about 100 um) surface layer. Zheng et al. prepared modified EP-based composites with different levels of carbon fiber doping [23]. The study showed that 6 wt% of carbon fiber doping is beneficial for improving the mechanical properties, thermal stability, and thermal conductivity of the EP-based composites. Hu et al. studied the thermal stability of polyaryletherketone (PAEK)-modified EP-based composites [24]. The results showed that as the mass fraction of thermoplastic PAEK increased, the diameter of rich spherical epoxy particles gradually decreased. The spherical structure helped to absorb energy, hindered crack propagation, and thus improved the fracture energy and toughness of the composite material. The heat resistance of PAEK/EP showed a trend of first increasing and then decreasing with the increase in the PAEK content. Li et al. studied the preparation method and mechanical properties of GF three-dimensional fabric-reinforced epoxy foam composite in order to reveal the reinforcing mechanism of the GF fabric [25]. It was shown that the introduction of three-dimensional fabric can significantly improve the strength of the composite under different load-bearing conditions.

Survey of the recent studies shows that a lot of work has been conducted on the high-temperature stability of the EP itself and the use of fiber fillers to enhance its mechanical properties. However, the number of studies on the effect of high-temperature aging on the mechanical properties of fiber-reinforced EP is limited to some extent. Therefore, the aim of this study is modification of a commercial EP by reinforcing it with 20% of GF to increase its heat aging resistance and mechanical properties. Heat aging effects on morphology, adhesion between GF and the EP matrix and mechanical properties at different temperatures are studied.

## 2. Materials and Methods

### 2.1. Materials

**Figure 1:**
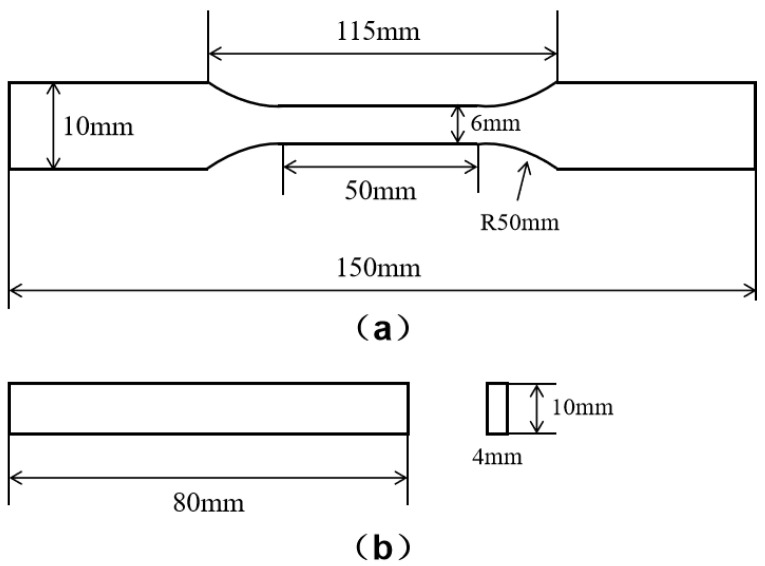
The dimensions of the dog bone sample used for tensile test (**a**) and the plate sample used for flexural test (**b**).

In this study, diglycidyl ether of bisphenol A (170–192 EEW) EP matrix and 3,3-diaminodiphenyl sulfone as a curing agent (Honghe Limited Corporation, Zigong, China) reinforced with 20% of GF with the average diameter of 10 μm (FR5301B-2000, Chongqing Polymer Composite International, Chongqing, China) were used. To prepare GF/EP composites, first, the EP was mixed with the curing agent. Then, a layer of GF was immersed into the still liquid epoxy resin, taken out, placed in the mold and maintained for curing. These operations were repeated for several layers stacked together to obtain a composite material with a laminar structure. Next, dog-bone-shaped and plate samples made of the GF/EP composite were prepared for mechanical tests. The samples were used to study heat aging effects on tensile and flexural properties. The dimensions of samples made for tensile measurements are shown in Figure 1a. The samples used for flexural tests had dimensions of 80 × 10 × 4 mm^3^ with a support span of 64 mm, as shown in Figure 1b.

### 2.2. Thermal Aging Treatment

Heat aging of the reinforced GF/EP samples was carried out in air atmosphere in the HZ-2004 furnace (Lyxyan, Dongguan, China) at different temperatures of 85, 100, 115, 130, and 145 °C for 180 h at each temperature. The samples were placed in the furnace after reaching the required temperature. Reference samples, i.e., not heat treated, were also prepared. In this study, we denote samples before the heat treatment as GF/EP-0 and samples after the heat treatment at a certain temperature as, for example, GF/EP-145, where 145 is the aging temperature of 145 °C.

### 2.3. Measurement Methods

#### 2.3.1. Mechanical Properties

To study damage effects of the GF/EP internal structure on the mechanical properties, tensile and flexural tests were carried out after different heat treatments. The dog bone samples were used to evaluate tensile properties using the ASTM D3039 standard. A mechanical tester MTS E45 (MTS, Eden Prairie, MN, USA) was used to conduct the tensile tests. The measurements were conducted at room temperature and at a cross-head speed of 50 mm/min. The gauge length region displacement was measured by a 50 mm length extensometer. After each heat treatment, five samples were measured to calculate averaged values of the tensile characteristics. In the experiment, tensile strain, tensile strength and elastic modulus were measured. Mechanical tester MTS E45 was also used to measure flexural properties of the composite. For these measurements, plate samples were used. The three-point-mode ASTM D7264 standard was employed. The cross-head speed was 1 mm/min. Five tests were performed to obtain averaged values of the flexural strength.

#### 2.3.2. Surface Morphology

To analyze the failure mechanism, morphology of the fractured surface of the sample was studied using scanning electron microscope (SEM) TESCAN MIRA III (TESCAN, Brno, Czech Republic) operated at 5 kV. The SEM samples were prepared from fractured samples after tensile measurements. Before the study, due to poor electrical conductivity of the GF/EP composite, the samples were coated with conductive carbon film using vapor deposition technique.

## 3. Experimental Results and Discussion

### 3.1. Tensile Properties

**Figure 2:**
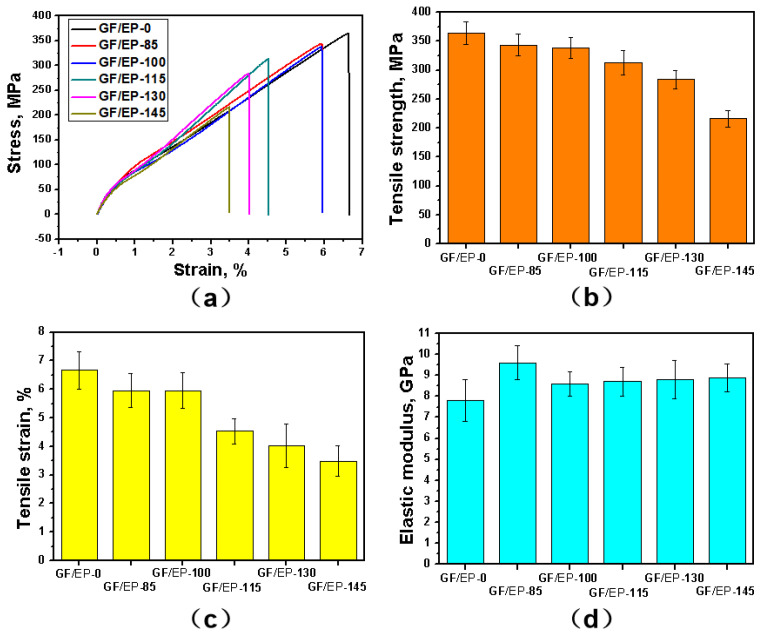
Tensile stress as a function of the strain measured after different heat treatments (**a**), tensile strength (**b**), strain (**c**) and elastic modulus (**d**) as functions of annealing temperatures measured for GF/EP composites after heat treatment.

One of the most important mechanical properties is tensile strength, which indicates the ability of material to resist forces that pull it apart. Figure 2 shows the effect of the heat treatment at different temperatures on the tensile characteristics of the GF/EP composites. The tensile stress as a function of the tensile strain measured after heat treatment of the GF/EP composite samples is shown in Figure 2a. All samples demonstrate nearly linear stress–strain curves according to Hook’s law of elastic deformation. The sample before thermal aging (GF/EP-0) shows a sharp decrease in tensile stress at the strain of 6.7%, indicating the failure of the sample. After heat treatment at 85 °C, the GF/EP-85 sample demonstrates a similar tensile stress–strain curve; however, both the strength and strain of the sample are smaller. At higher aging temperatures in the range of 100 and 145 °C, the samples demonstrate similar linear stress–strain curves, followed by failure of the sample and further decrease in the strength and strain. The tensile strength, tensile strain, and elastic modulus as functions of the aging temperature are shown in Figure 2b–d. In Figure 2b, the tensile strength of the samples after heat treatment shows a decrease along with increasing aging temperature. The values of the tensile strength of the samples GF/EP-0, GF/EP-85, GF/EP-100, GF/EP-115, GF/EP-130, and GF/EP-145 are 364.4, 343.6, 338.1, 313.5, 283.8, and 216.4 MPa, respectively. Faster decrease in tensile strength at higher temperatures indicates accelerated structural degradation of the GF/EP composite. Figure 2c shows tensile strain as a function of the aging temperature. The values of tensile strain of the samples GF/EP-0, GF/EP-85, GF/EP-100, GF/EP-115, GF/EP-130, and GF/EP-145 are 6.7%, 6.0%, 5.9%, 4.5%, 4.0% and 3.5%, respectively. Figure 2d shows that within the experimental uncertainty, the elastic modulus is independent of the aging temperature. Therefore, one can conclude that heat treatment results in degradation of the GF/EP composite ductility. This effect can occur due to destruction of the internal structure of the GF/EP composite such as cross-linking and chain scission of the initial linear molecular structures of the EP matrix during thermal aging. Moreover, a simultaneous increase in crystallinity of the material takes place, which also increases the brittleness of the composite. Degradation of the internal structure of the EP due to increase in crystallinity and cross-linking depends on the aging temperature and treatment time. It is also known that interaction between GF and EP controls the mechanical properties of the GF/EP composites. Therefore, it can be concluded that another reason of degradation of mechanical properties can be a decrease in the interfacial adhesion force between GF and the EP matrix. Such degradation can occur due to oxidation of the EP matrix during the heat treatment or due to difference in coefficients of thermal expansion (CTEs) of the GF and EP. The oxidation of the EP itself can also have an effect on its crystallinity.

### 3.2. Failure Analysis

**Figure 3:**
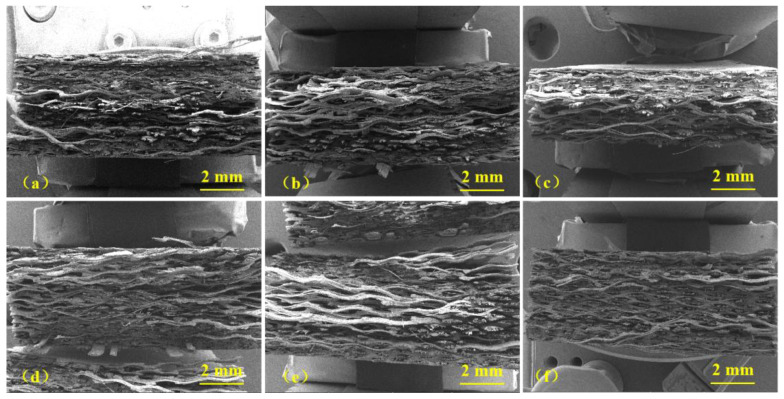
The SEM images of the fracture morphology taken at a low magnification after heat treatment at different temperatures of the composites: (**a**) GF/EP-0, (**b**) GF/EP-85, (**c**) GF/EP-100, (**d**) GF/EP-115, (**e**) GF/EP-130, and (**f**) GF/EP-145.

**Figure 4:**
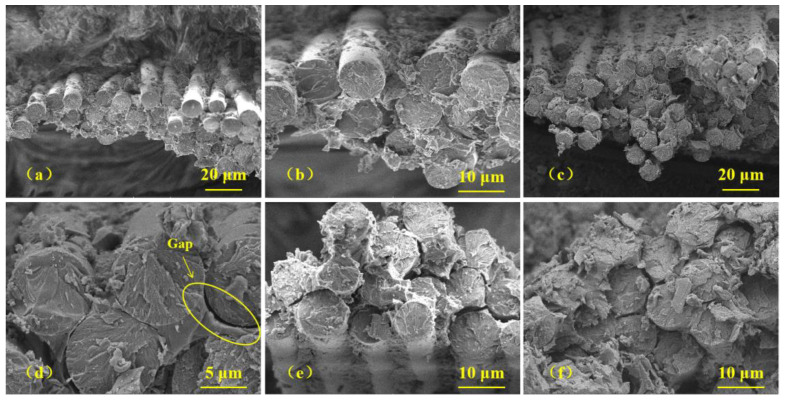
The same as in Figure 3 at higher magnification. Samples: (**a**) GF/EP-0, (**b**) GF/EP-85, (**c**) GF/EP-100, (**d**) GF/EP-115, (**e**) GF/EP-130, (**f**) GF/EP-145.

The cross-section SEM images of the GF/EP composites before and after heat treatment at different temperatures are shown in Figure 3 and Figure 4. Figure 3 shows the overall cross-section of the fractured surface of the composites at a low magnification. A laminar morphology of the structure is well observed. Each layer is composed of GF embedded in the EP matrix. There is no evident crack initiation or propagation, which means that the GF/EP composite sample has high toughness. Moreover, no obvious difference in overall fracture morphology between the composite samples aged at different temperatures is observed. Therefore, to study the failure behavior of the aged composite samples, it is necessary to analyze SEM images at a higher magnification. These SEM images of the GF/EP composites after fracture in tensile test are shown in Figure 4. GF wires embedded in the EP matrix now are very well visible. In Figure 4a, fracture morphology of the sample GF/EP-0 before heat treatment is shown. It can be seen that each GF is coated well with the EP matrix, which proves highest tensile strength and explains good adhesion between GF and the EP matrix. After heat treatment at 85 and 100 °C, the GFs are still observed as embedded in the EP matrix, as shown in Figure 4b,c, respectively. The GFs are visible as pulled out of the EP matrix. Therefore, a good adhesion of the EP matrix with GFs up to aging temperature up to 100 °C is observed. However, after heat treatment at 115 °C, as shown in Figure 4d, numerous slits between GFs and the EP matrix can be observed, which suggests degradation of the adhesion force due to heat treatment. Figure 4e shows that at higher temperatures of 130 and 145 °C, there is more evident detachment of the EP matrix from the GF surface, suggesting further degradation of the adhesion between components. To explain this heat treatment effect, we propose that the main reason for detachment of the GF from the EP matrix is the difference in their CTEs. The EP CTE is in the range of 7–9·10^−5^ K^−1^ [26], and for the GF, we can use the CTE of the α-quartz, which is in the range of 7.1–13.1·10^−6^ K^−1^ and depends on the crystal orientation [27]. It can be seen that there is an order-of-magnitude difference in the CTEs of EP and GF. Thus, at a relatively low aging temperature, thermal stress appears between these two materials due to the difference in CTEs, whereas at a higher aging temperature, the thermal stress damages the GF/EP interface, which eventually results in degradation of mechanical properties.

### 3.3. Flexural Properties

**Figure 5:**
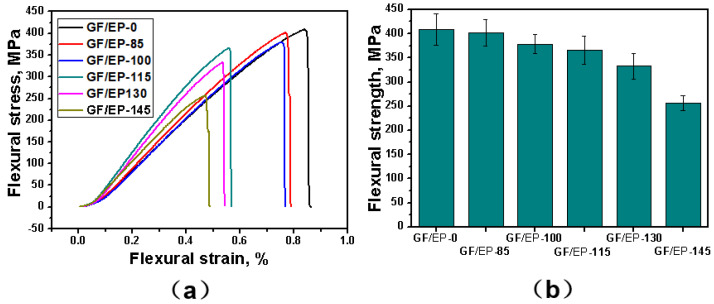
The flexural stress as a function of the strain (**a**) and the flexural strength of the GF/EP samples heat treated at different temperatures (**b**).

Flexural stress–strain curves are shown in Figure 5a. All curves have two parts. First, a linear part demonstrates the elastic deformation. The second sublinear part represents the elastic–plastic deformation followed by sample fracture. The non-heat-treated GF/EP-0 sample has the highest flexural strength and strain. After heat treatment, the samples GF/EP-115 and GF/EP-130 show faster increase in the stress as a function of strain. Influence of the temperature of the aging on flexural strength is shown in Figure 5b. The flexural strength decreases along with the increase in aging temperature from 408.4 to 255.7 MPa. The minimal flexural strength is demonstrated by the sample heat treated at the highest temperature. Degradation of the flexural strength can also be explained by the increase in brittleness of the GF/EP composites after heat treatment due to cross-linking and chain scission of the initial molecular structure of the composite and decrease in the interfacial adhesion force between GF and EP caused by oxidation of the EP matrix or difference in the GF and EP CTEs. The general temperature trend of the flexural strength is in agreement with that of the tensile strength, shown in Figure 2.

## 4. Conclusions

Layered composites of diglycidyl ether of bisphenol A EP reinforced with a 20% GF were prepared. Tensile and flexural stress–strain curves of the GF/EP composite after aging tests in the temperature range of 85–145 °C in air wrre measured. As the aging temperature increases from 85 to 145 °C, the tensile and flexural strength demonstrate decrease from 364.4 to 216.4 MPa and from 408.4 to 255.7 MPa, respectively. However, the elastic modulus of the samples (~8.5 GPa) is almost independent of the aging temperature. The SEM images of the fracture morphology indicate that samples are degraded by thermal aging from the brittle manner. This effect can occur due to destruction of the internal structure of the GF/EP composite such as cross-linking and chain scission of the initial linear molecular structures of the EP during thermal aging. SEM images also reveal a separation of the GF and the EP matrix, which is caused by a decrease in the adhesion force between GF and EP and results in the observed deterioration of mechanical properties. We explain the decrease in the interfacial adhesion between GF and EP by the fact of oxidation of the EP matrix and the difference in the GF and EP coefficients of thermal expansion, which is one order of magnitude. This work can be useful to understand the effect of thermal aging on the stability of the mechanical properties of the GF/EP composites.

## Data Availability

Not applicable.
